# Language Dominance Affects Bilingual Performance and Processing Outcomes in Adulthood

**DOI:** 10.3389/fpsyg.2018.01199

**Published:** 2018-07-26

**Authors:** Eloi Puig-Mayenco, Ian Cunnings, Fatih Bayram, David Miller, Susagna Tubau, Jason Rothman

**Affiliations:** ^1^School of Psychology and Clinical Language Sciences, University of Reading, Reading, United Kingdom; ^2^Department of Language and Culture, UiT the Arctic University of Norway, Tromsø, Norway; ^3^Departament de Filologia Anglesa i Germanística, Universitat Autònoma de Barcelona, Barcelona, Spain

**Keywords:** language dominance, Negative Concord Items, Differential Object Marking, early bilinguals, Catalan/Spanish

## Abstract

This study examines the role of language dominance (LD) on linguistic competence outcomes in two types of early bilinguals: (i) child L2 learners of Catalan (L1 Spanish-L2 Catalan and, (ii) child Spanish L2 learners (L1 Catalan-L2 Spanish). Most child L2 studies typically focus on the development of the languages during childhood and either focus on L1 development or L2 development. Typically, these child L2 learners are immersed in the second language. We capitalize on the unique situation in Catalonia, testing the Spanish and Catalan of both sets of bilinguals, where dominance in either Spanish or Catalan is possible. We examine the co-occurrence of Sentential Negation (SN) with a Negative Concord Item (NCI) in pre-verbal position (Catalan only) and Differential Object Marking (DOM) (Spanish only). The results show that remaining dominant in the L1 contributes to the maintenance of target-like behavior in the language.

## Introduction

A large body of studies involving early childhood bilinguals examine the development of linguistic competence during the acquisition process itself, often focusing on how bilingual acquisition is qualitatively similar or different to monolinguals during the developmental period of language learning (see Meisel, 2011; Serratrice, 2013; Nicoladis, 2018 for a review). Furthermore, studies concerned with adult second language acquisition or first language attrition largely focus on similar processes; however, they do so with inherently different contexts concerning age of onset and other deterministic variables (see Rothman and Slabakova, 2017; White, 2018; Wulff and Ellis, 2018; Yilmaz and Schmid, 2018 for updated reviews from various paradigmatic approaches). The focus, thus, is on the acquisition of another language starting in adulthood and the ensuing developmental consequences, as in the case of attrition, on the maintenance of previously acquired languages.

A notable exception to the trends in the above literature is the work on heritage speaker (HS) bilingualism (see Montrul, 2008, 2016; Rothman, 2009; Benmamoun et al., 2013; Kupisch and Rothman, 2016; Polinsky, 2018). To date, the focus within HS bilingualism has been to examine adult steady-state grammars of (at least) the minority (heritage) language acquired in early childhood. The heritage language is one of the HS's L1s, either acquired simultaneously with the societal majority language (2L1) or as the unique L1 in the case of child L2 acquisition whereby immigration occurs before or at school age (roughly 5–6 years old). Thus, HSs are a subtype of native speaker (Rothman and Treffers-Daller, 2014). This is interesting given that studies generally reveal that adult HS grammars reflect both dominance in the majority language (i.e., whether a simultaneous L1 or child L2 for the HS) and degrees of non-monolingual-like variability in the heritage L1 (see Montrul, 2008, 2016; Benmamoun et al., 2013).

The typical HS outcomes are, at first glance, surprising in light of child 2L1 and child L2 studies that generally demonstrate greater conformity, whether in qualitative similarities in development and/or ultimate attainment (see for review Meisel, 2011; Haznedar, 2013; Chondrogianni, 2018). After all, HSs tested as adults are the outcomes of 2L1 or child L2 acquisition. As such, we are left to wonder why they differ to such a degree in adulthood from the seemingly successful trajectory that research on child bilingualism suggests they were on (Kupisch and Rothman, 2016). In recent years, several researchers have suggested that HSs' grammatical outcomes in adulthood likely highlight distinctive acquisition paths, reflecting the individual realities of personal, minority language/bilingual situations for variables that become more deterministic in later childhood (e.g., Putnam and Sánchez, 2013; Kupisch and Rothman, 2016). In other words, in addition to effects of L1 attrition and/or arrested development at the individual HS level, linguistic and extra-linguistic variables conspire to change the path of HS grammatical development and, thus, explain the default trend of considerable variation both between HSs and monolinguals, as well as other HSs. The emerging literature has highlighted the following variables, among others: (1) the quality of input affected by language contact (L1 attrition of the older generation); (2) the lack of literacy in the heritage language; (3) the influence of formal properties (features) of the majority language, altering the formal HS learning task; and (4) being outside a bilingual community representing true diglossia. All of these variables reduce opportunities to use the minority language and receive/uptake (quantity/quality) input (e.g., Putnam and Sánchez, 2013; Kupisch and Rothman, 2016; Bayram et al., 2017; Karayayla and Schmid, 2017; Karayayla, 2018).

In the vast majority of work on heritage bilingualism to date, the default context is one of a distinct majority language that subsumes the minority one in all aspects of societal distribution (e.g., only the heritage community is bilingual in the languages under investigation whereby education is typically in the majority language) and there is a palpable imbalance of prestige between the two languages. It is this situation itself that promotes the abovementioned imbalance in extra-linguistic variables. If the unequal distribution of these extra-linguistic variables across various HS groups or individuals factors into the unique outcomes of HSs (Lloyd-Smith et al., submitted), then we should see monolingual-to-bilingual differences significantly diminish or be eradicated in the adult outcomes of 2L1 speakers and especially child L2 bilinguals when the context for bilingualism is more favorable. This should be especially true when the society itself is bilingual in the same languages.

The case of Catalonia is an ideal environment to put the above to test as successful bilingualism is the default in this setting, inclusive of the purposeful efforts in place in the education system to ensure that all young people are formally literate and educated in both languages. The fact that there is near universal success in Catalan-Spanish bilingual outcomes does not negate the fact that the order of acquisition of both languages can vary across individuals, and that depending on where in Catalonia one grows up it could be said that one or the other is more dominant. Moreover, successful bilingualism at the community level does not preclude cross-linguistic influence in developing bilingual grammars. Looking at how differences might obtain even in such a context, and whether this correlates/varies with order of acquisition and other measures of relevant dominance (patterns of use) in one or the other language, can augment the heritage speaker literature more generally. Minimally, showing what is similar and distinct both between our bilinguals here and more typical HS outcomes can reveal what is likely to differ between monolingual and child bilingual outcomes in adulthood universally vs. what obtains independently as the byproduct of the less-than-ideal bilingual environments HSs tend to grow up in.^1^

In the present study, all bilinguals are formally trained in literacy in both languages. We provide data from two groups of Catalan-Spanish bilinguals who were born and raised in Osona, Catalonia where dominance in Catalan is the default.^2^ The first group comprises child L2 learners of Spanish (L1-Cat-L2-Sp) and the second group comprises child L2 learners of Catalan (L1-Sp-L2-Cat). The present study is also one of a select few that tests each bilingual group in both languages, which is needed to understand more fully how the languages of a bilingual interact and how this might differ across bilingual groups depending on factors such as the ones that differentiate our bilingual groups from those pertaining to typical HS environments.

Given this relatively unique environment, one can find bilinguals who are more dominant in one or the other language while highly proficient and literate in both. It is not uncommon to find a child L2 learner of Catalan in Catalonia who remains dominant in their L1 (Spanish), unlike the typical case of immersed child L2 learners. What is especially interesting about Osona is that the minority (Spanish) and the majority (Catalan) languages of the immediate regional society, which should matter most, are the opposite in the national context. This variable will be considered pertaining to the generalizability of the results.^3^ However, Catalonia is certainly not the only context in the world where this applies. Beyond contributing to the literature by offering a study that examines somewhat different conditions for the outcomes of a case of child L2 bilingualism in adulthood (as well as potential consequences to their L1), we endeavor to show how capitalizing on the unique positioning of variables that contexts like Catalonia present by default can inform important questions of theoretical relevance. Minimally, isolating some of these extra-linguistic variables has the potential to explain individual variation across bilingual speakers of the same two languages, even when both languages are readily available in the environment and supported via education.

We investigate two subtle phenomena in Spanish and Catalan: (1) the co-occurrence of Sentential Negation (SN) with a Negative Concord Item (NCI) in pre-verbal position, allowed in Catalan yet disallowed in Spanish and (2) Differential Object Marking (DOM), obligatory in Spanish but not part of the Catalan grammar. We chose these phenomena because they are claimed to be sensitive to variation in the adult grammars of childhood bilinguals (Montrul, 2004; Déprez et al., 2015) in other contexts.

## Theoretical background

Our chosen properties are of particular interest because they allow us to look at whether order of acquisition and language dominance play a role in the expansion of the distribution of a specific linguistic domain. Negative Concord Items (NCIs) in Catalan have a wider distribution [with and without sentential negation (SN)] than in Spanish (without SN). The distribution of DOM in Catalan and Spanish also presents differently, whereby Spanish has a wider distribution of DOM than Catalan. Though variable across dialects, DOM in Spanish *par excellance* (i.e., across dialects) is obligatory in certain cases, such as marking accusative [+animate/+specific] objects. Indeed DOM is subject to semantic and discourse constraints in particular contexts (e.g., as it interfaces with modality, indicative vs. subjunctive in embedded clauses); however, in the domain of DOM we focus on there is no such considerations affecting its use. In other words, it is a morphosyntactic reflex of obligatory (accusative) case marking. DOM is more restricted in Catalan and is ungrammatical in the Spanish-canonical position of [+animate/+specific] objects in their base-generated position. In both cases, the smaller distribution is subsumed by the language with the larger distribution: (a) all contexts in which DOM exists in Catalan exist in Spanish, but Spanish has more obligatory DOM contexts and (b) all contexts where Spanish NCI is allowed hold true for Catalan, although Catalan also allows it with SN. And so, assuming that influence will proceed from a subset to a superset, choosing these two domains allows us to look without prejudice for one language over the other into whether CLI will obtain accordingly in relatively balanced bilingualism (no differences related to relative dominance), or if CLI is conditioned by relative dominance in one or the other language.

### Negative concord items (NCI_S_) in catalan and spanish

NCIs have been argued to be negative Universal Quantifiers (Haegeman and Zanuttini, 1991; Zanuttini, 1991), positive Polarity Items (Laka, 1990), negative indefinites (Suñer, 1995), and non-negative indefinites (Zeilstra, 2004; Tubau, 2008). Herein, we adopt Zeilstra's (2004) analysis of NCIs specifically for Catalan and Spanish while considering some modifications offered by Espinal and Tubau (2016).

Both Catalan and Spanish are Negative Concord (NC) languages. NC languages are typified by two main varieties: strict NC Languages, in which the sentential negation (SN) is always obligatory, as in Romanian; and non-strict NC languages, in which the sentential negation is obligatory when the NCI is in post-verbal position and disallowed when the NCI is in pre-verbal position, such as in Spanish. Note that there is a third option that is universally marked, which is essentially a weak version of the strict NC language option described above. In such cases, the negative marker is possible with a pre-verbal NCI but not obligatory. Among the members of the Romance family, Catalan seems to be the only language that allows for optionality of the negative marker when the NCI is in pre-verbal position (Quer, 1993; Vallduví, 1994; Espinal, 2000; Tubau, 2008). All of this can be seen in the grammaticality of (1a-1b) and (4a-4b), the ungrammaticality in (2a-2c) and the variation in grammaticality of (3a-3b) and (4a-4b).


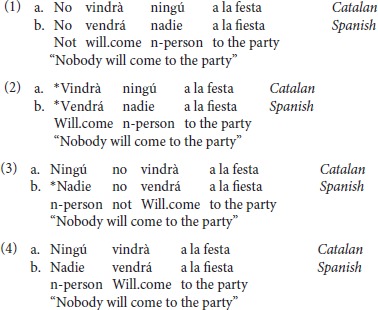


### Differential object marking

DOM is the overt morphological expression used by some languages to mark Case on some accusative objects. Spanish is known to be a DOM language (e.g., Leonetti, 2004; López, 2012). Unlike Spanish, Catalan presents a less clear case;^4^ however, it is well attested that in both Standard Catalan and the Central Catalan dialect, which are the dialects relevant to our bilingual groups herein, DOM is not expected (Escandell-Vidal, 2009; GIEC, 2016).

Rodríguez-Mondoñedo (2007) suggests that there are two important dimensions which help determine the marking of the object: animacy and specificity.^5^ As pointed out by Leonetti (2004), animacy has been labeled as the dominant factor. If we use these two dimensions, there are four possible scenarios for objects: [+specific/+animate], [+animate/–specific], [–animate/+specific] and [–animate, –specific]. In Spanish, the object is obligatorily marked when the object is [+specific, + animate] as in (5a-b).


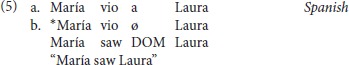


When the object is [–animate/+specific] or [–animate/–specific], then the object is obligatorily unmarked. The case of [+animate/–specific] can be marked, this this depends on various semantic and discourse features that we highlight here for the sake of being complete. As we only focus on [+animate/+specific] contexts in which the marker is obligatory and, to our knowledge, not subject to dialectal variation as other subtypes are, we will not comment further on the inherent variation of DOM cross-dialectically.

Importantly, the distribution of DOM in Standard Catalan and Central Catalan is more restricted than in Spanish. For example, in Catalan [+animate/+specific] full DP objects are left unmarked (compare 8a-b). Thus, the experiments herein contain full DPs.


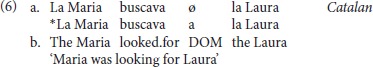


However, the fact that the marker does not appear in this context does not mean that DOM is non-existent in Catalan, a point to which we return in the discussion when we discuss the input. As reported in (GIEC, 2016), DOM is required when the [+animate/+specific] is a full pronoun or in cases where the full DP object is found in focalized constructions. In sum, the above illustrates that DOM occurs in certain contexts in Catalan, but, crucially, does not occur in the context under investigation, which entails that its distribution is somewhat more restricted than in Spanish.

### Studies on catalan-spanish bilingualism

Although there is a line of research that has looked at developmental patterns in Catalan-Spanish bilinguals (e.g., Bel, 1996, 2001, 2003; Bosch and Sebastían-Gallés, 2001; Guijarro-Fuentes and Marinis, 2009; Simonet, 2011, 2014; Guijarro-Fuentes, 2012; Illamola, 2015; Perpiñán, 2017), there are relatively few studies that have examined Catalan/Spanish bilingualism outcomes in adulthood. In that respect, Perpiñán (2017) stands out as a noteworthy study examining the effects of early bilingualism in adulthood in the domain of non-personal clitics in Catalan that are lacking in Spanish [i.e., the partitive clitic (*en*) and the locative clitic (*hi*)]. Her results show that the group of Spanish-dominant speakers were significantly less sensitive to instances of ungrammaticality than the Catalan-dominant speakers. This is an expected, though significant, result. It is often the case that bilingual knowledge differs significantly from the anticipated monolingual outcome. However, in a context like Catalonia where relatively balanced bilingualism is likely, and both languages are supported at all levels, it is reasonable to hypothesize that bilingual grammars would differ less from monolinguals than in other cases of bilingualism. Indeed, this expectation has some evidence. Recall that the Catalan-dominant group is also bilingual, yet conforms to monolingual norms significantly more and thus reveals that dominance, even in a society where access to both languages is ubiquitous, matters.

Studies like Perpiñán (2017) are significant because they show more of the same, that is, they highlight the effects of bilingualism that exist despite a context that is maximally supportive for success and, crucially, seem to suggest that dominance—and not proficiency *per se*—matters. Consequently, it is clear that bilingualism effects are real, meaning some differences in bilingual grammars obtain because of bilingualism itself (Sorace, 2011) and not merely because of extra-linguistic considerations such as poor access to input, low prestige of a weaker language, etc., that define the reality of many, or perhaps most, of the realities of individual bilinguals. However, the fact that bilingualism itself, even under ideal contexts, can invite monolingual base-line differences—bilingualism is not multiple instances of monolingualism in the same mind/brain (Grosjean, 1989)—does not mean that lack of such a supportive environment and the entailed beneficial byproducts of it would not further exaggerate monolingual-bilingual differences. In other words, what would the speakers in Perpiñán (2017) look like if they grew up in a less supportive bilingual environment, such as a typical HS environment? On the basis of this work, we expect some cross-linguistic influences in our Catalan-Spanish bilinguals, but, like Perpiñán (2017), we expect them to be subtle differences and not subject to a large amount of inter-speaker variation as is the default when typical HSs are tested.

### DOM and NCIs in catalan-spanish bilingualism

Although there has been considerable work in recent years examining the acquisition of DOM in L2 Spanish (e.g., Farley and McCollam, 2004; Montrul, 2004; Bowles and Montrul, 2008; Guijarro-Fuentes and Marinis, 2009; Guijarro-Fuentes, 2012), and how it appears in HS Spanish grammars in North America (e.g., Montrul, 2004; Montrul and Bowles, 2009; Montrul and Sánchez Walker, 2013) to various degrees of successful convergence, we are aware of only one study that examines it in the context of Catalan-Spanish bilingualism (Guijarro-Fuentes and Marinis, 2009). In this study, the authors showed that Catalan-Spanish sequential bilinguals, although outperforming English learners of L2 Spanish, were still considerably different from Spanish monolinguals in the sense that they over-accepted the accusative makers in contexts where they were not grammatical. Guijarro-Fuentes and Marinis (2009) make no mention of having tested for language dominance (LD); however, given the context and the fact that they are home speakers of Catalan, it is fair to assume that if they were not balanced bilinguals, LD for the group would be in Catalan. From their results, we know that grammatical sensitivity to Spanish DOM can be affected by the more restricted domain of DOM in Catalan. What we do not know, however, is if the restricted DOM in Catalan can be affected (expanded) by Spanish in the opposite direction of LD. This latter point is addressed by the present bi-directional study.

Contrary to the case of DOM, there is a dearth of available studies looking at NCIs from an acquisition perspective in the Catalan-Spanish bilingual literature. However, experimental research in syntax has been done to corroborate current theoretical descriptions in both languages. Déprez et al. (2015) examine the interpretation of pre-verbal NCI when occurring with Sentential Negation (SN) in Catalan. They examined whether the co-occurrence of the SN would trigger Double Negation (DN) readings of the NCI as opposed to NC readings. Their findings suggest that the default readingof a pre-verbal NCI in Catalan with the SN is a generally an NC one, which is not possible in Spanish (Déprez et al., 2015; Espinal et al., 2016).

## Research questions and predictions

The main overarching research question that motivated the present study was:

- What role do order of acquisition and language dominance have—independent of overall linguistic proficiency—in the competence and performance of early child bilinguals tested in adulthood?

As is true of all specific research, overarching questions must be packaged in testable ways, examining specific domains of grammar in specific sub-groups of participants under appropriate contexts as proxies. And so, question (a) can be asked as (b):

b. What is the respective role of order of acquisition and dominance in Catalan and Spanish regarding the competence and performance outcomes of NCIs and DOM among early child bilinguals tested in adulthood?

Our hypotheses are:

c. Language dominance matters. Cross-linguistic influence (CLI) from Catalan-to-Spanish and Spanish-to-Catalan is *a priori* possible for both groups. Perhaps, irrespective of dominance, some CLI will be noted. We also predict that greater degrees of CLI might correlate to relative dominance, in which case there would be significant differences across the two groups. We also hypothesize that the domain of grammar matters. CLI is conditioned by the comparative status of the properties in the two grammars; CLI will influence expansion in the grammar with a more restricted distribution. This means we expect emerging optionality in Spanish NCIs and/or expansion of DOM in Catalan contexts where it is prohibited via influence of the larger distribution in the other grammar, but not vice versa. That is, Catalan may lose optionality in NCI interpretation or Spanish may lose DOM in canonical contexts not supported by Catalan. We further predict that there could be differences across the two domains of grammar, whereby NCIs are either not affected or they are less affected because Catalan's larger grammar reflects optionality which contains the Spanish obligatory option, compared to the case of DOM where Spanish, the larger grammar, reflects obligatory use of DOM in unattested contexts of Catalan.

## Methodology

### Participants

We tested two groups of participants who differ in their order of acquisition and their reported language use and exposure. We included only participants whose proxy for dominance, assessed by means of reported use and exposure via the Language Experience and Proficiency Questionnaire (LEAP-Q) (Marian et al., 2007), indicated accordance between their L1 (Spanish or Catalan) and their dominance in adulthood. Although the default assumption of HS bilingualism in general is that dominance in adulthood will be in the L2, we are interested in knowing what effects bilingualism has in the case that one can and does remain dominant in their L1 even if, like the typical HS situation, it is not the preferred, majority language of the bilingual situation. Thus, in an effort to not muddy the waters, we examined bilinguals who were balanced in proficiency across the two languages, yet each group remains dominant in their L1. The first group of participants consists of Spanish-Catalan bilinguals who were exposed to Spanish from birth and Catalan at schooling age: L1Sp-L2Cat speakers (*n* = 23). Though the schooling system is generally in Catalan and the language of the environment is Catalan, they reported high levels of use of and exposure to Spanish.^6^ The second group is comprised of Catalan-Spanish bilinguals who were exposed to Catalan at home and whose first significant exposure to Spanish was at school age: L1Cat-L2Sp speakers (*n* = 21).

All participants were vetted to ensure fullfilment of the inclusion criteria: (1) Catalan/Spanish bilinguals with no other native languages, (2) minimum proficiency in any foreign languages,^7^ (3) high native scores in both Catalan and Spanish proficiency tests, and (4) residence in the geographical (Osona) area where data were collected (Central Catalan dialect). Spanish proficiency was measured through the DELE, which is standardly used as a measure of proficiency in the field (e.g., Montrul and Slabakova, 2003; Slabakova and Montrul, 2003; Bruhn de Garavito and Valenzuela, 2008; Slabakova et al., 2012). Catalan proficiency was measured using a part of the *Certificat Superior de Llengua Catalana* implemented by the *Centre de Normalització Lingü*í*stica*.

The Leap-Q was used to assess overall language use and exposure, which we used as a proxy for dominance. We also examined answers from the Catalan version of the Leap-Q questionnaire. We first looked at their responses of the questionnaire:^8^ question 1 (dominant language), question 3 (exposure to each language), question 5 (use of both languages); and their responses in the questions for each language: question 2, 4, and 5 (exposure in different environments). Such questions probed self-reported percentages of use and exposure to each language, as well as assessing amount of exposure on a scale from 1 to 10 (1 = not much exposure; 10 = a lot of exposure). A participant was categorized as dominant in one language or the other when two or three of the following conditions were met: (a) reported exposure in one language was higher than the other, (b) reported use in one language was higher than the other, and (c) the self- rated exposure to one of the language was higher than the other. Table 1 provides the participant profiles after the inclusion criteria had been applied.

**Table 1:** Details of the participants.

	**L1Sp-L2Cat (*N* = 23)**	**L1Cat-L2Sp (*N* = 21)**
Mean age	22	20
Proficiency in Catalan	34/40	36/40
Proficiency n Spanish	46/50	45/50
Dominant Language	Spanish	Catalan
Mean (%) exposure to Spanish	59%	18%
Mean (%) exposure to Catalan	41%	82%
Mean (%) use of Spanish	66%	85%
Mean (%) use of Catalan	34%	15%
Rate (1-to-10) of exposure to Spanish	6.5/10	2.5/10
Rate (1-to-10) of exposure to Catalan	3.5/10	7.5/10

This study was carried out in accordance with the recommendations of Research Ethics Committee. The protocol was approved by the School of Psychology and Clinical Language Science's Research Ethics Committee at the University of Reading. All subjects gave written informed consent in accordance with the Declaration of Helsinki.

### Tasks

Participants took part in two separate experimental tasks: an off-line Grammaticality Judgement Task and a non-cumulative, moving window Self-Paced Reading (SPR) Task in both languages.^9^ Presentation by language was counter-balanced: half of the participants were asked to do the Catalan experiments first and vice versa. All the tasks were delivered using IBEX FARM software and the experiments were done in a controlled lab environment.

#### Grammaticality judgement task

All participants completed two Grammaticality Judgement Tasks: one in Spanish and another in Catalan. Each task consisted of 48 items which were distributed across six conditions (four target conditions + two filler conditions) with eight items per condition. The four target conditions are described below.

Condition (a) (NCI+SN) consisted of sentences with a NCI [*nadie*, Sp or *ningú*, Cat; “*nobody*” in pre-verbal position followed by the negative marker *no*. This structure is ungrammatical in Spanish, but grammatical in Catalan [see examples in (7a-b)]. The items in condition (b) (NCI-SN) were target sentences containing an NCI without the negative marker—a structure which is acceptable in both Catalan and Spanish. [See examples in (8a-b)].

Condition (c) (+DOM) consisted of items with a [+animate, +specific] marked DP object by the Accusative Marker “*a*.” In Spanish, this is grammatical, whereas in Central Catalan and Standard Catalan it is ungrammatical. Condition d) (–DOM) consisted of items in [+animate, + specific] without the accusative marker. This is grammatical in Catalan and ungrammatical in Spanish. See examples (9a-b) and (10a-b).

The sentences in these two tasks were judged on a 6-point Likert scale where 1 was completely odd and 6 was completely natural. There was also an option of “I'm not sure.” Participants were instructed to answer as fast as possible and to leave aside any prescriptive judgements by rating the sentences according to their own intuitions. There were eight practice items, after which the experimental items started.


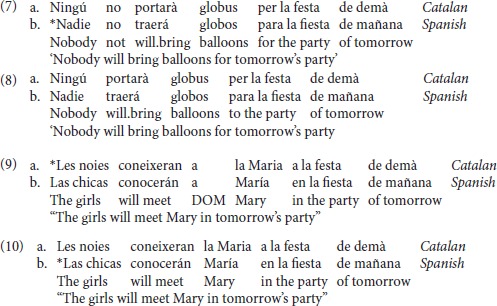


#### Self-paced reading task

The Self-Paced Reading Task was also administered in each language and used the same four experimental conditions [(a) NCI+SN, (b) NCI–SN, (c) +DOM and (d) –DOM], each of which contained eight items, in addition to four filler conditions (*n* = 64). The filler conditions consisted of sentences with similar structures but without the occurrence of NCIs or DOM. Each item was divided into regions of interest which were then used to examine reaction times and spill-over effects. An example of this division can be seen in (11a-b) below


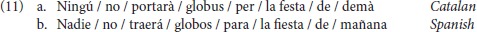


We created two lexical sub-contexts such that the sentences did not become repetitive: half of the experimental items had vocabulary related to a party and half of them to a market. Examples of experimental items can be seen in (7–10) above as we used similar sentences to those in the GJT. Participants were instructed to read the sentences at a normal pace and respond to comprehension questions. They were instructed to do the first three items and to ask any questions, after which the experiment started with six distractor items, then the 64 items were presented in a random fashion.

## Results

### Grammaticality judgement tasks

#### Descriptive analysis

Table 2 presents the Grammaticality Judgement data in both Catalan and Spanish from the two experimental groups: L1Cat-L2Sp (*n* = 21) and L1Sp-L2Cat (*n* = 23) for the two properties and all the conditions. In order to conduct the statistical analysis, the responses in the 6-point Likert scale were converted using a binary coding: responses from 1 to 3 were coded as rejection “0” and responses from 4 to 6 were coded as acceptance “1”^10^

**Table 2:** Raw count of acceptance by condition for the two properties and two groups^a^.

	**Catalan**		**Spanish**	
**NCI CONDITIONS**				
	**NCI**+**SN**	**NCI-SN**	**NCI**+**SN**	**NCI-SN**
L1Cat-L2Sp (*n* = 21)	127/168	141/168	36/168	147/168
L1Sp-L2Cat (*n* = 23)	147/184	163/184	35/184	160/184
**DOM CONDITIONS**				
	**–DOM**	**+DOM**	**–DOM**	**+DOM**
L1Cat-L2Sp (*n* = 21)	112/168	106/168	72/168	162/168
L1Sp-L2Cat (*n* = 23)	129/184	114/184	51/164	168/184

a *The total percentage of “I do not know” responses is 0.014%*.

The results for the NCI+SN and NCI–SN conditions in Table 2 show the expected distribution as predicted by the theoretical analysis, that is, acceptance of both conditions in Catalan, which confirms the optionality of the SN *no*. In Spanish, there is a strong acceptance of the NCI–SN condition and rejection of the NCI+SN, confirming the lack of optionality of sentential negation with preverbal NCIs. Recall that all DOM targets in Spanish only require the accusative *a* marker and therefore, what is reported as –DOM is when the *a* is missing (ungrammatical in Spanish, yet the only grammatical in Catalan) and +DOM is when the *a* is present (grammatical in Spanish and ungrammatical in Catalan). The results for the –DOM condition in Catalan indicate target-like performance for both groups, however, both groups have high acceptance of the +DOM condition in Catalan (ungrammatical). When the participants are tested in Spanish, they each show target-like acceptance of the +DOM sentences, but, in the ungrammatical condition (-DOM), they also show a slight over-acceptance.

#### Statistical analysis

To further investigate the findings, we conducted linear mixed effects logistic regression analyses of the responses in the R environment (R Core Team, 2016), by using the lme4 package (Bates et al., 2015). Generalized mixed effects models were fit to the binomial response data. The data for the two properties under investigation were analyzed separately in each language, thus, we used separate models. Each model included fixed effects of condition (Model1: NCI+SN, NCI+V; Model2: –DOM, +DOM), group (L1Cat-L2Sp, L1Sp-L2Cat,) and their interaction. Fixd effects were sum-coded as −0.5/0.5 and each model included by-participant and by-item random intercepts and slopes for the repeated measures variables. In the case of significant interactions, planned comparisons investigated effects of group within the same condition using the multcomp package (Hothorn et al., 2008). The summaries of the omnibus models are presented in Tables 3, 4.

**Table 3:** Generalized mixed effects models for the NCI property in the two different datasets (RL: L1Cat-L2SP, NCI+SN).

	**Catalan data**		**Spanish data**	
	**Estimate (SE)**	***p***	**Estimate (SE)**	***p***
(Intercept)	1.094 (0.28)	<0.001	−1.338 (0.44)	<0.001
Group	0.251 (0.25)	0.316	−0.181 (0.44)	0.488
Condition	0.726 (0.43)	0.092	3.278 (0.55)	<0.001
Group: Condition	0.334 (0.42)	0.418	0.413 (0.46)	0.322

**Table 4:** Generalized mixed effects models for the DOM property in the two different datasets (RL: L1Cat-L2SP, –DOM).

	**Catalan data**		**Spanish data**	
	**Estimate (SE)**	***p***	**Estimate (SE)**	***p***
(Intercept)	1.815 (0.28)	<0.001	−1.092 (0.44)	<0.001
Group	−0.114 (0.34)	0.738	−0.216 (0.38)	0.039
Condition	−1.627 (0.34)	<0.001	2.877 (0.54)	<0.001
Group: Condition	0.595 (0.39)	0.136	0.022 (0.39)	0.954

For the NCI data the effect of condition was significant for the Spanish data only, in the absence of any significant interactions. This confirms that both groups allowed both conditions in Catalan and that both groups significantly preferred the NCI+V condition in Spanish. For the DOM data, there was a significant main effect of condition in both Catalan and Spanish, with a preference for the grammatical condition in each language.

The results show that both groups have target-like grammars in both Catalan and Spanish with respect to the NCIs. They all allow for optionality in the co-occurrence of the NCI (ningú) and Sentential Negation (no) as expected and they do not allow this optionality in Spanish. With regards to the DOM conditions, both groups prefer the grammatical condition in each language +DOM in Spanish and –DOM in Catalan, but both groups also show an unexpected over-acceptance of ungrammatical conditions in both languages.

### Self-paced reading tasks

Comprehension accuracy was calculated to ensure that participants were reading the sentences and paying attention to the task. The mean accuracy for the L1Cat-L2Sp group is 93.04% in Spanish and 95.61% in Catalan. The rates of comprehension accuracy for the L1Sp-L2Cat were 92.30% in Spanish and 94.31% in Catalan. This indicates that the participants paid attention to the task.

**Figure 1:**
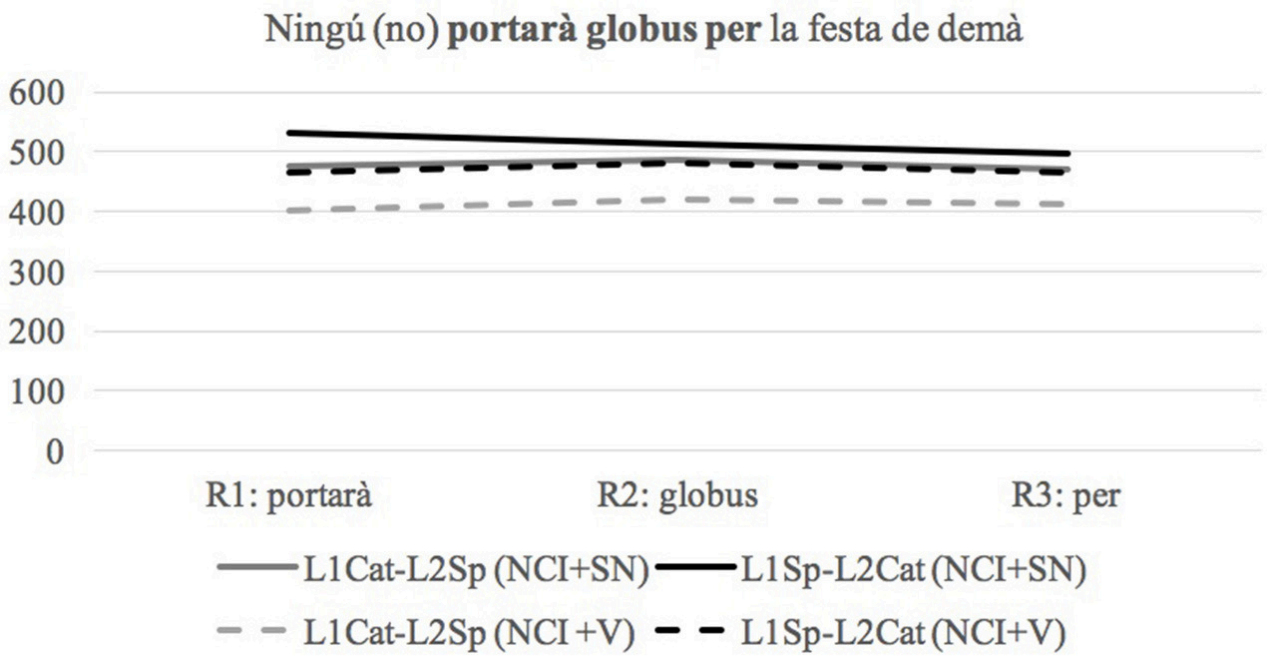
Line graph of Reaction Times (RTs) in milliseconds (ms) in the regions of interest in the NCI conditions when tested in Catalan.

The analysis focuses on the three regions following the Critical Region to check for any slowing down effects (i.e., spill-over effects). This is done due to the fact that for two of the four conditions, the Critical Region was an empty region (absence of Sentential Negation or absence of the accusative marker). The reaction times (RTs) for each condition and each language were analyzed separately (NCI+SN, NCI–SN, +DOM, –DOM) using linear mixed effects models, using the same coding scheme as for the offline data. We used raw RT as opposed to residual because the critical comparisons are the same across conditions rendering residualization not necessary. The regions of interest were of the same length, both groups are equally bilingual (scoring at ceiling in proficiency in both languages), each bilingual group is highly literate in both languages and most crucially, there is purposefully no monolingual control comparison from which we might expect a general difference in reaction time. Figure 1 shows the mean RTs (ms) for the three regions of interest and each group in the NCI conditions when the groups are tested in Catalan.

The three models revealed no significant main effects or interactions (see Table 5), which indicates that both the L1Cat-L2Sp and the L1Sp-L2Cat groups allow optionality with respect to the co-occurrence of pre-verbal NCIs and Sentential negation in Catalan.

**Table 5:** Linear models for the NCI property Catalan (RL: L1Cat-L2SP, NCI+SN).

	**R1**		**R2**		**R3**	
	**Estimate (SE) *t*-value**	***P***	**Estimate (SE) *t*-value**	***p***	**Estimate (SE) *t*-value**	***p***
Intercept	452.1 (32.7) 13.809	<0.001	463.9 (32.1) 14.459	<0.001	394.5 (26.6) 14.822	<0.001
Condition	−81.6 (50.4) 1.619	0.105	−45.55 (40.7) −1.118	0.263	−85.6 (45.5) −1.882	0.069
Group	77.3 (46.7) 1.654	0.09	66.9 (52.06) 1.654	0.198	66.8 (38.1) 1.753	0.079
Condition^*^Group	−60.6 (42.1) 1.439	0.149	−07.6 (31.6) −1.439	0.809	−82.3 (52.5) −1.566	0.117

**Figure 2:**
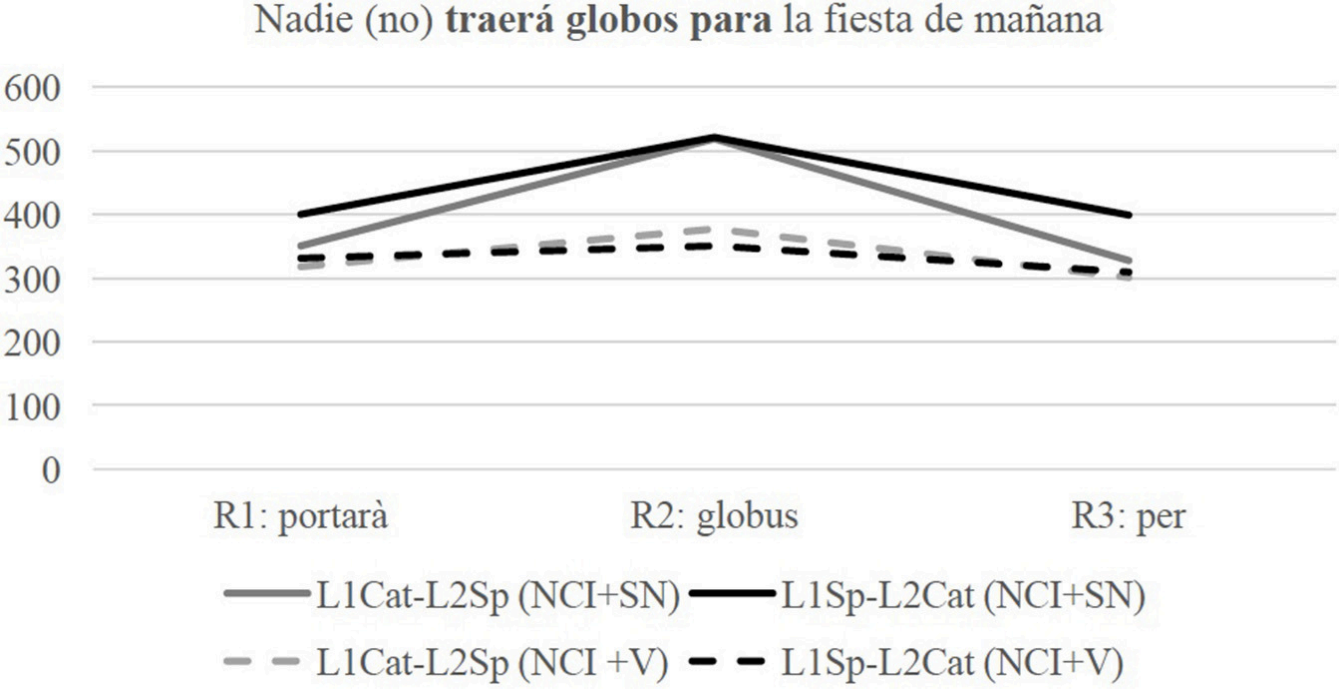
Line graph of Reaction Times (RT) in milliseconds (ms) in the regions of interest in the NCI conditions when tested in Spanish.

When they are tested in Spanish in these same conditions, the picture that emerges is different (see Figure 2).

As seen in Table 6, the only significant main effect was the one on condition in R2, showing that both groups are significantly slower in the second region of interest of the NCI+SN (ungrammatical in Spanish) than in the NCI+V (grammatical). The results show that both groups are sensitive to the morphosyntactic violation of pre-verbal NCIs co-occurring with Sentential Negation in Spanish.

**Table 6:** Linear models for the NCI property in Spanish (RL: L1Cat-L2SP, NCI+SN).

	**R1**		**R2**		**R3**	
	**Estimate (SE) *t*-value**	***p***	**Estimate (SE) *t*-value**	***P***	**Estimate (SE) *t*-value**	***P***
Intercept	399.2 (38.6) 10.329	<0.001	452.5 (17.3) 26.164	<0.001	335.3 (18.5) 18.092	<0.001
Condition	1.45 (60.5) 0.025	0.979	−190.6 (19.1) −9.995	<0.001	−46.4 (28.2) −1.693	0.092
Group	113.69 (77.3) 1.471	0.141	−26.1 (34.3) −0.759	0.447	51.8 (34.6) 1.496	0.134
Condition^*^Group	50.81 (120.9) 0.420	0.420	−44.1 (36.9) −1.192	0.233	−58.9 (49.79) −1.192	0.236

**Figure 3:**
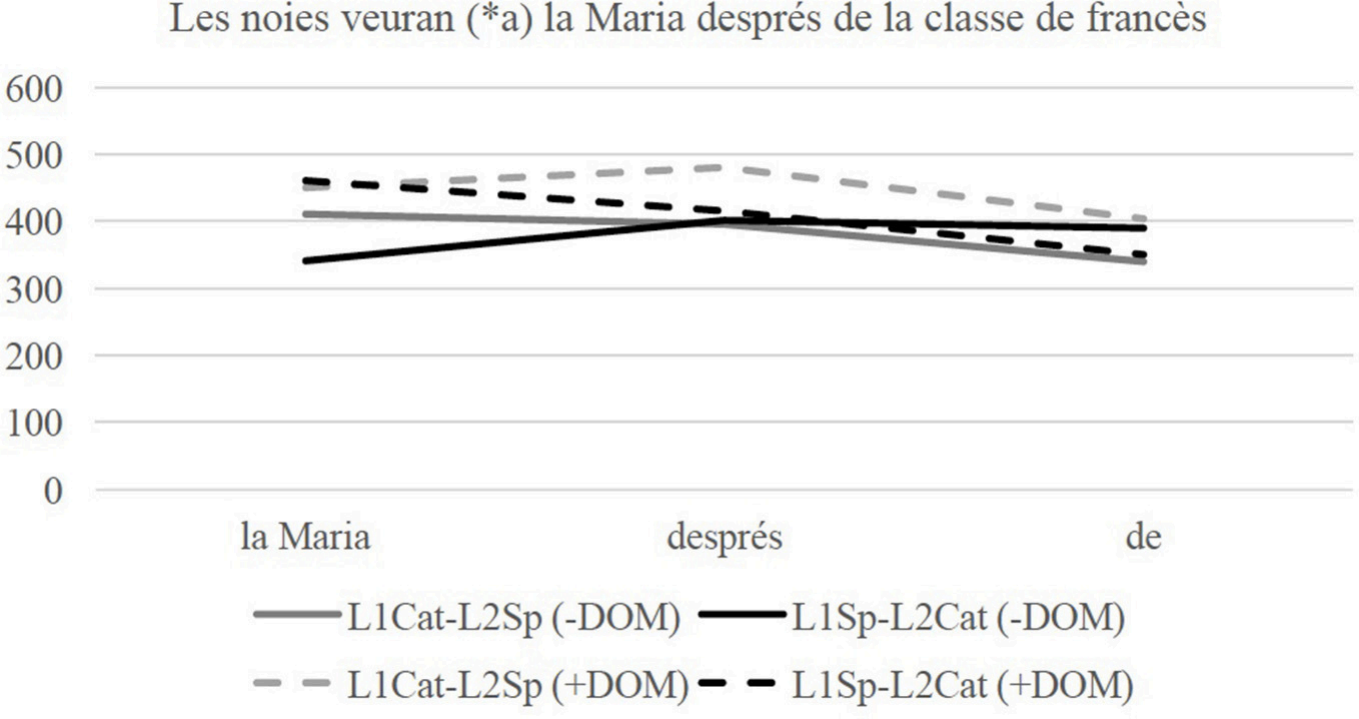
Line graph of reaction times (RTs) in milliseconds (ms) in the regions of interest in the DOM conditions when tested in Catalan.

Turning to the DOM conditions, Figure 3 illustrates the Catalan data.

The statistical results in Table 7 show that there is a significant interaction of Group^*^Condition in the third region of interest. The results indicate that the L1Cat-L2Sp group does not show sensitivity to the morphosyntactic violation of the +DOM condition and the L1Sp-L2Cat group shows sensitivity to the –DOM condition, being significantly slower in the first (*p* = 0.025) and third region (*p* < 0.001). This shows that the L1Cat-L2Sp group has optionality in their grammars because they allow sentences with the accusative marker and without it in Catalan and that the L1Sp-L2Cat disallows the absence of the accusative marker, potentially showing influence from Spanish onto Catalan.

**Table 7:** Linear models for the DOM property in Catalan (RL: L1Cat-L2SP, –DOM).

	**R1**		**R2**		**R3**	
	**Estimate (SE) *t*-value**	***p***	**Estimate (SE) *t*-value**	***p***	**Estimate (SE) *t*-value**	***p***
Intercept	454.2 (29.7) 15.265	<0.001	455.1 (27.7) 16.411	<0.001	371.4 (22.7) 22.674	<0.001
Condition	−19.9 (48.3) −0.415	0.678	−15.5 (49.9) −0.310	0.756	15.3 (26.33) 0.581	0.561
Group	65.9 (41.4) 1.583	0.113	45.4 (29.8) 1.515	0.129	1.44 (26.36) 0.054	0.956
Condition^*^Group	−98.4 (44.1) −2.232	0.025	−196.9 (35.8) −5.496	0.388	−121.18 (35.5) −3.410	<0.001

**Figure 4:**
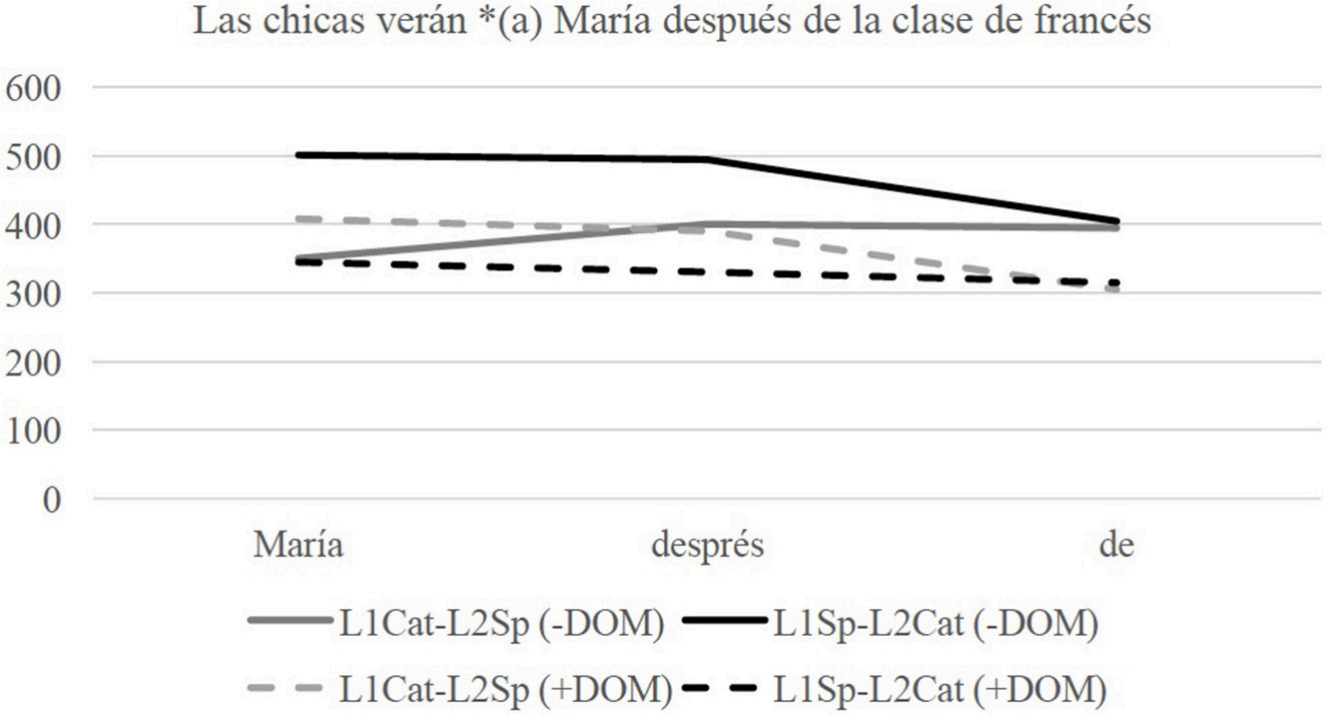
Line graph of Reaction Times (RT) in milliseconds (ms) in the regions of interest in the DOM conditions in Spanish.

The following Figure 4 shows the Spanish Data in the DOM conditions.

The statistical models in Table 8 show a significant interaction of Group^*^Condition in Region 1, reflecting that the L1Cat-L2Sp group is significantly slower in the +DOM condition (*p* < 0.001) and the L1Sp-L2Cat group is significantly slower in the –DOM condition. In the third region, there is also significant interaction of Group^*^Condition, the L1Sp-L2Cat group is significantly slower in the –DOM condition. Overall, the results show that the group of L1Sp-L2Cat group have target-like grammar and that the L1Cat-L2Sp group show sensitivity to the expected grammatical condition, thus, their grammar shows influence from Catalan with respect to this phenomenon in Spanish.

**Table 8:** Generalized linear models for the DOM property in Spanish (RL: L1Cat-L2SP, –DOM).

	**R1**		**R2**		**R3**	
	**Estimate (SE) *t*-value**	***p***	**Estimate (SE) *t*-value**	***p***	**Estimate (SE) *t* = value**	***p***
Intercept	406.7 (43.8) 9.273	<0.001	365.9 (21.7) 16.846	<0.001	355.3 (18.8) 18.805	<0.001
Condition	15.3 (26.3) −0.619	0.535	−101.3 (30.7) −3.297	<0.001	−63.4 (31.5) −2.010	0.027
Group	44.1 (87.7) 0.502	0.615	75.3 (42.4) 1.774	0.076	4.89 (35.1) 0.139	0.889
Condition^*^Group	−232.3 (35.5) −1.365	<0.001	−84.5 (58.5) −1.443	0.148	27.5 (56.4) 0.487	0.625

## Discussion and conclusions

In this section, we bring the results together in summary. As there is a significant amount of data to be considered, we begin with a brief overview of the most interesting results. Starting with the NCI conditions, as can be seen in Table 9, irrespective of modality (offline vs. online) and the language of testing, each group's performances are consistent with having distinct representations for both languages that conform to what is formally described of Spanish and Catalan. As a result we can safely say that order of acquisition and/or relative dominance in one or the other language brings nothing to bear for this domain of grammar, at least for these sets of bilinguals, a point to which we return below.

**Table 9:** Summary of the results for the NCI conditions, where (✓) refers to expected performance based and (✘) does not.

	**L1Sp-L2Cat**		**L1Cat-L2Sp**	
**Spanish**	NCI+SN (GJT) ✓	NCI–SN(GJT) ✓	NCI+SN (GJT) ✓	NCI–SN(GJT) ✓
	NCI+SN (SPR) ✓	NCI–SN (SPR) ✓	NCI+SN (SPR) ✓	NCI–SN (SPR ✓)
**Catalan**	NCI+SN (GJT) ✓	NCI–SN(GJT) ✓	NCI+SN (GJT) ✓	NCI–SN(GJT) ✓
	NCI+SN (SPR) ✓	NCI–SN (SPR) ✓	NCI+SN (SPR) ✓	NCI–SN (SPR) ✓

Turning to the DOM conditions, the picture is less clear. We have some within group mismatches in performance across modalities as well as inter-group across language and modality-group, as can be appreciated visually in Table 10 below. Our focus is definitively not on any comparisons to monolinguals, but rather on a fairer bilingual-to-bilingual group comparison (e.g., Ortega, 2010, 2013; Rothman and Iverson, 2010; Hopp and Schmid, 2013) where L1 and L2 status is switched in a mirror-image way and proficiency is held constantly high in. That said, we do highlight below where group diverges from expected monolingual norms, as described in the literature, with some insights as to why this might be. Attempting to compare the bilinguals to monolingual control groups would have been difficult, in part since it would be virtually impossible to find a Catalan monolingual control group and thus it would have been unbalance if we were to offer only a Spanish one. At first glance, however, it is useful to highlight, as we predicted could occur, that CLI can be conditioned by the domain of grammar itself, a point to which we will return in greater detail below.

**Table 10:** Summary of the results for the DOM conditions, where (✓) refers to expected performance based and (✘) does not.

	**L1Sp-L2Cat**		**L1Cat-L2Sp**	
**Spanish**	+DOM (GJT) ✓	–DOM (GJT) ✘	+DOM (GJT) ✓	–DOM (GJT) ✘
	+DOM (SPR) ✓	–DOM (SPR) ✓	+DOM (SPR) ✓	–DOM (SPR) ✘
**Catalan**	+DOM (GJT) ✘	–DOM (GJT) ✓	+DOM (GJT) ✘	–DOM (GJT) ✓
	+DOM (SPR) ✘	–DOM (SPR) ✘	+DOM (SPR) ✘	–DOM (SPR) ✓

Looking at the quadrant on the top-left side of the table shaded in green, that is when L1-Sp-L2-Cat bilinguals are tested in their L1, Spanish, we see that for the –DOM conditions—where the accusative marker *a* is not present although it is grammatically obligatory—the GJT revealed influence from Catalan, their L2. This is not terribly surprising in light of previous literature that has shown DOM to be highly vulnerable in bilingual contexts (e.g., Guijarro-Fuentes and Marinis, 2009; Montrul et al., 2015) even for the context we used—purposefully because dialectal variation that can otherwise obtain for DOM in other contexts does not apply. However, it is not clear at what level this Catalan influence rests—e.g., if such reflects a representational difference in their mental grammars—precisely because in the SPR task the same participants do show a clear sensitivity to the very same ungrammatical condition. If it were truly the case that these speakers' grammars did not have the functional architecture of Spanish DOM in their grammar, we would expect that they would be equally insensitive to DOM grammaticality issues in both modalities. The fact that the processing measure shows sensitivity that is potentially obscured in the offline behavioral measures alone might be because the processing measures are more likely to tap into implicit knowledge (e.g., Jegerski, 2014; Keating and Jegerski, 2015). Therefore, we would not conclude based on a coupling of the two modalities that these L1-Sp-L2-Cat bilinguals have non-monolingual-like representations for DOM, but rather that the offline task shows a more methodological performance based difficulty. This same pattern, where processing measures indicate better competence than offline behavioral measures, has been shown recently for other types of Spanish bilinguals, namely more traditional HSs in North America (e.g., Villegas, 2014; Jegerski et al., 2016).

Shifting to the bottom-left quadrant of the table shaded in blue, that is when the L1-Sp-L2-Cat bilinguals are tested in Catalan, they show over-acceptance of sentences with +DOM (ungrammatical in Catalan) in the GJT and they do not show sensitivity to the morphosyntactic violation in this condition in the SPR either. Because there is performance conformity across modalities, we take this as especially strong evidence that the underlying reason for both performances is one and the same, that is, representational in nature. The performance seems to suggest that Spanish is influencing their Catalan. In turn, their performance in Catalan as summarized in Table 10 is further evidence for what we argued in relation to the representation of this domain in their Spanish grammar. Recall that they appeared to have some issues marking –DOM as ungrammatical despite having no issues accepting +DOM as grammatical and being sensitive to the –DOM violation in RT. We concluded that the processing measure reflected their competence more accurately. Their performance on the Catalan condition thus seems to strengthen this claim precisely because one could only reasonably expect (or explain) evidence of Spanish DOM transfer in Catalan if indeed they had an intact DOM representation from their other grammar. There is also a modality asymmetry in their Catalan performance for the same domain, that is –DOM, however, this seems to be the mirror image of their performance in Spanish. In Catalan, they perform just fine in the –DOM condition, which entails accepting as grammatical sentences that do not have an overt *a* case marker, in the offline measure only. With the same condition in the online measure, they show a sensitivity (they slow down) where they should not, suggesting that they are sensitive to a grammatical violation that should not obtain in Catalan but does in Spanish. We would like to suggest that the offline measure potentially reflects a “yes” bias, they simply did not reject something provided to them and that the online measure reflects more their grammatical representation, which we take to be influenced from Spanish. To the extent that this is on the right track, it again provides further evidence for intact DOM representations in Spanish.

These results, related back to our research question that probes the relationship that language use and exposure exercises on linguistic competence/performance in both languages of early child bilinguals, suggest that language use and exposure play a role in determining the directionality of cross-linguistic influence.^11^ Recall that this set of participant was categorized as having high use and exposure to Spanish even though they live in a Catalan-dominant area. We conclude that contrary to other typical cases of Spanish Heritage Speaker bilingualism, the access to high quality and quantity of input to the minority language of the immediate context (i.e., Spanish)—by means of language use and exposure on top of education—is a key factor to preventing cross-linguistic interference from the majority language of the immediate context (i.e., Catalan).

Turning to the L1-Cat-L2-Sp bilinguals, we focus our attention to the quadrant on the top-right of the table shaded in orange. Particularly notable is the fact that they do not judge the –DOM conditions in Spanish as categorically ungrammatical (GJT), nor do they show appropriate sensitivity to the ungrammaticality in this condition. However, in the +DOM conditions, they show target-like performance in the GJT and SPR tasks. Since it is the case that these bilinguals do not reliably reject nor show sensitivity in RT to sentences in Spanish without the accusative *a* marking when the object is [+ animate, +specific], the canonical condition under which DOM is required, yet have no issues accepting sentences that have it in the same context, we might conclude that they indeed have a representation for DOM in their mental Spanish grammars, but, unlike the other group and other sets of Spanish natives described in the literature, DOM seems optional as opposed to obligatory. Such a conclusion might be strengthened by the latent patterns in their performance. That is, in both the –DOM and +DOM they are consistent in their performances across offline and online modalities.

Turning to the final quadrant in the bottom right shaded in yellow, that is, when the L1-Cat-L2-Sp participants are tested in their native Catalan, we see that although they prefer sentences without DOM (grammatical in Catalan) by rating them as more acceptable than sentences with DOM (ungrammatical in Catalan), they do accept +DOM sentences at a non-trivial rate. In the online data, these speakers show no sensitivity in –DOM conditions, as expected, however, they do not show sensitivity to the grammatical violation of +DOM conditions in Catalan. Taken together, this also suggests that their grammars allow for optionality with respect to DOM, which goes in line with Chondrogianni (2018) claim that DOM in Catalan is starting to appear in varieties of Catalan which traditionally do not allow for it. Optionality in their Spanish grammar, thus, can be explained by influence from Catalan on their Spanish precisely because their Catalan shows the same degree of optionality. As it relates to the question of language dominance (LD), again we see that LD affects cross-linguistic influence in these highly proficient bilinguals. At first glance, because there is optionality that would not be expected *per se* of a monolingual native Catalan grammar (to the extent that there are any) it was not clear that LD, in this case Catalan influence, was unambiguously demonstrated or at least as clearly as it was for the Spanish dominant group. However, since we have shown that the optionality in Spanish is reflected also in the Catalan of these same speakers it seems reasonable to understand the optionality in Spanish as influence of Catalan as represented in these bilinguals.^12^ Thus, we have evidence of LD affecting cross-linguistic influence in both groups.

It is interesting to ponder why out of the two domains of grammar tested, both of which differ across the languages, only one shows cross-linguistic influence, albeit patterning differently, in both target groups. It is possible that the issues with DOM are idiosyncratic to DOM itself. Recall that DOM seems to be challenging in all instances of heritage speaker bilingual acquisition (see e.g., Montrul et al., 2015). Moreover, we should keep in mind that the accusative case marker itself is phonologically reduced and potentially not overly salient. Furthermore, DOM reflects a large degree of variation across Spanish dialects and even individual speakers. Because our bilinguals, however, are all exposed to Peninsular Spanish where DOM is consistent in the core context we isolated (López, 2012) and given that the [+ animate, +specific] is not subject anyway to much variation dialectically or individually, we attempted to control for the general variation within this grammatical domain, which was chosen precisely because it had been shown to be problematic for more typical HSs. Keeping in mind our research questions then and under the hypothesis that less variation would obtain in our context of societal bilingualism as compared to more traditional HS situations, examining a domain such as DOM, as compared to other properties, could then go a long way to inform us about what is vulnerable in bilingualism even when many variables that likely affect HS performance are more favorably proportioned. And so, why all of these factors might contribute to why DOM is a vulnerable property for bilingual variation in general, they do not seem to be overcome as they are for monolinguals even in an environment where all opportunity has been given for our bilinguals to perform like monolinguals. This should of course not be surprising and certainly bespeaks nothing evaluative about our bilinguals herein, why would they or how could they perform exactly like monolinguals, if only because they are simply not monolinguals? However, given the differences across the two groups that grow up under similarly favorable environmental conditions, there does seem to be some evidence to suggest that order of acquisition/language dominance matters for the outcomes of development in this domain. And so, relating more directly all that we have seen across the two domains of grammar to our two research questions, it seems that LD matters for some domains of grammar more than others, even when bilinguals are more or less balanced as related to overall proficiency in the languages and when this is maximally supported by a bilingual environment. If the same pattern holds for future studies of a similar nature, then looking at the adult outcomes of such groups as we have done here might couple together with more traditional HS populations to inform linguistic theory more generally. As Polinsky (2016, 2018) has nicely argued and supported with data recently, certain domains of grammar are invulnerable to bilingual effects even in the minority language of HSs who are severely imbalanced in dominance whereas others are highly sensitive to bilingual effects. Our data then support her general claim (see Tsimpli, 2014 for similar arguments), showing that some properties of grammar are still vulnerable to bilingual effects while others are not even in the opposite case, that is, when there is extremely high proficiency in both languages and the day-to-day environment of the bilingual promotes both languages. Together, such data can tell us what is more and less core related to language in general.

As promised above, it is worth coming back to the case of NCIs and ponder why there is no CLI noted at all, that is, conditioned or not by order of acquisition/dominance, different from the case of DOM. The case of NCIs is interesting by comparison to DOM, since only the former relates to optionality in the “larger” grammar. Catalan permits the Spanish sole, obligatory spell-out [the use of the NCI without Sentential Negation (SN)] but optionally allows for double negation spell-out without the canceling of semantic negation (as would be the case in Spanish if an NCI co-occurred with SN). And so, there is no direct competition of an obligatory nature between the two grammars, as is the case with DOM where an obligatory use of DOM constitutes an ungrammatical extension of DOM in Catalan. Therefore, it could be the case that this tension “optionality” vs. “obligatoriness” plays a further conditioning role for CLI. In a sense, the grammars might be less likely to affect one another when what is at stake in not a contradiction in the obligatory construction of a grammatical structure. The subtleties involved, in other words, are actually not so subtle. The case of NCI might stand out across the two languages as more salient precisely because Catalan optionality coincides with a very specific domain of distribution in which it reflects an interpretation that is unavailable in Spanish.

As a closing point of discussion, it is worth considering whether or not our speakers are indeed HSs of a specific sub-type or if it would indeed be best to not apply that label to them. In an effort to not open up Pandora's box on this potential issue, we were neutral in distinguishing traditional HSs from our bilinguals herein and mainly because it hardly matters for our immediate points. We could be neutral because there is no denying the fact that our bilinguals are quite different in non-trivial ways from Spanish HSs studied in North America. But those differences alone do not necessarily mean that they are both not HSs, yet of distinct types (see Putnam et al., 2018 for similar argumentation). Although more traditional HSs do not remain dominant in their HL because their environments essentially preclude this and it is seemingly a given that HSs will show, on a gradient, differences from expected monolingual baselines (but see Kupisch and Rothman, 2016), a lack of difference in these regards should not be used as a criterion to disqualify someone as a HS. Doing so would only make sense under a deficit model of HS bilingualism whereby the label HS has somehow become synonymous with deficiency par excellence. With many others (e.g., Putnam and Sánchez, 2013; Kupisch and Rothman, 2016; Bayram et al., 2017; Putnam et al., 2018), we definitively reject such a view. Allowing for the present bilingual groups to be considered as a specific subtype of HSs, precisely because they meet all the neutral inclusion criteria of several non-deficit approaches definitions widely adopted in the literature, for example, Rothman (2009). And so, evidence from highly balanced HSs, if the label is appropriate to apply to our L1-Sp-L2-Cat group, could go a long way at counterbalancing the HS as an incomplete acquirer viewpoint. Our L1-Sp-L2-Cat participants grew up in a household where both parents had moved to rural Catalonia and are not native speakers of Catalan, Spanish is their exclusive L1 and the only language spoken in their homes when they were young children and continues to be the family language. Crucially, the majority language of the immediate environment they grow up in is not their home language, but rather (for them) an L2 (Catalan), which they became significantly immersed in only upon going to school. This means that Spanish is their native L1, unlike the L1-Cat-L2-Sp group for whom Spanish is clearly an L2. It is also true that in this environment successful bilingualism and support for such is omnipresent and, thus, the possibility to maintain and further develop Spanish is different than other typical cases of HSs. Spanish has a higher prestige and is more accessible than it is in the USA, however, in this specific part of Catalonia there is no question that Spanish is not the majority language of the society (see Illamola, 2015). The increased opportunity to conserve dominance in Spanish does not disqualify our HSs from being HSs, it merely naturally creates an environment in which we can observe the relative weight of key variables that are different from Spanish HS situations in other environments and could not otherwise be teased apart. And so, why should our population not reflect a sub-type of HS? We leave this discussion for future work that takes advantage more and more of what comparisons of traditional HSs and bilinguals like ours can show when the minority language, in this case Spanish, is able to be held constant.

## Author contributions

EP-M is the main author. JR is the second main author and lab director. IC worked on the statistical analysis. FB, DM and ST worked on the conceptualization, design and implementation of the study.

### Conflict of interest statement

The authors declare that the research was conducted in the absence of any commercial or financial relationships that could be construed as a potential conflict of interest.

## Footnotes

^1^Following neutral definitions for HS inclusion such as (Rothman, 2009) where deficit outcomes are not part of the criteria of defining factors of HSs, one could convincingly make an argument that children of parents who immigrate from monolingual Southern Spain and raise their children even in rural Catalonia as (virtual) monolingual Spanish speakers until they go to school are indeed a specific subtype of HSs. Of course, they would be exposed tangentially to Catalan, just like Spanish HSs are to English in the ubiquitously studied case of Spanish HS bilingualism in the US, before schooling starts. Not being significantly exposed to the other (societal majority language) is in fact even more possible in a place like Catalonia where everyone is bilingual, such that each individual a child encounters can effortlessly switch to the language the child prefers. Indeed, the societal status of the languages and all this entails for use and exposure differ significantly between the two contexts we are comparing, but these differences are exactly what we capitalize on as they permit a teasing out of variables otherwise not possible. Whether or not such speakers are in fact accepted as HSs by all is not important for the purposes of our argumentation. In the case they are not, we can only hope that the reason does obtain because of the general success of Catalan-Spanish bilingualism itself. HS bilingualism does not necessarily entail lack of success (Kupisch and Rothman, 2016), which is immediately clear if one accepts that our Spanish L1-Child L2 Catalan group should count as a subtype of HSs.

^2^Language dominance and its measurement have been widely debated (e.g., Bialystok, 2007; Montrul, 2015; Schmeißer et al., 2015; Silva-Corvalán and Treffers-Daller, 2015; Unsworth, 2015). In this study, by dominance we refer to patterns of preferred use and usage frequency in daily life following Unsworth's (2015) suggestion that language exposure/use patterns might be taken as a proxy of LD. As such one should not infer anything with regard to proficiency per se. As stated and will be quantified below, all participants are highly proficient, performing on the standardized measures for both languages with no statistical difference. Following Montrul (2015) and Schmeißer et al. (2015), we do not assume a direct relationship between dominance and proficiency per se, even if in unbalanced bilingual environments there tends to be correlations. As discussed in Perpiñán (2017) and quantified below, Catalan-Spanish bilinguals tend to be highly proficient in both languages, thus, correlating proficiency and language dominance in this context of balanced bilingualism might not prove useful.

^3^Although the majority of people in Catalonia are bilinguals speaking both languages (99% of the population speak and understand Spanish and 96.5% of the population speak and understand Catalan, Idescat, 2013). In this study, we targeted an area where Catalan is clearly the majority dominant language of the environment (73% of Catalan in the daily use in this area as opposed to 43% in Catalonia as a whole, Idescat, 2013). The reader is referred to Illamola (2015) for an in-depth presentation of the sociolinguistic patterns and the use of both Catalan and Spanish in the specific town (Manlleu, Osona) where the data were collected.

^4^DOM is present in some varieties of Catalan (potentially stemming from cross-linguistic influence issues related to Spanish as well), e.g., Balearic and Valencian Catalan (Escandell-Vidal, 2009; GIEC, 2016), however, DOM is definitively not part of the dialects spoken by the participants included in our study.

^5^The notion of specificity has been widely debated in the literature. We take a widely accepted notion that specificity expresses a semantic property of the element that determines the referent of the element in a particular way (see Farkas, 1995; von Heusinger, 2002; von Heusinger and Kaiser, 2003; Leonetti, 2004; López, 2012; for a more detailed description and analysis of specificity).

^6^It is crucial to recall here that our claim of the clear-cut nature of Catalan being the majority language relates to the specific location, central rural Catalonia. Such a claim would not be so evidently true in large Metropolitan areas, such as Barcelona. For example, Sorolla (2009) shows that children whose native language (home language) is Spanish tend not to use Catalan as much, even with peers, despite being educated in Catalan primarily. This is not so surprising given the demographics of such a large metropolitan area, the very reason we decided to test in rural Catalonia where the incidence of ethnic Catalans as discussed in footnote 3 (see work by Illamola, 2015).

^7^We would have wanted to exclude participants with knowledge of foreign languages, but English is mandatory in the system and they all had, at least, minimal exposure to it.

^8^See the corresponding questions in the English version of the Leap-Q questionnaire.

^9^The raw data of the experimental task will be made available by the authors, without undue reservation, to any qualified researcher.

^10^We present the results collapsed in a binary coding for ease of exposition and to make a clearer distinction between grammatical and ungrammatical. A similar analysis was conducted on the 1-to-6 scale data and the overall picture was the same.

^11^The two languages under investigation are two closely related systems and thus, this might have had an effect on triggering cross-linguistics effects. However, cross-linguistic influence in the DOM we have investigated has also been reported when Spanish is in context with other less related language, such as English, in context of Spanish as a Heritage Language in the US (e.g., Montrul, 2004; Montrul and Bowles, 2009; Montrul and Sánchez Walker, 2013) or Spanish as a non-native language (e.g., Farley and McCollam, 2004; Bowles and Montrul, 2008) in North America (e.g., Montrul, 2004; Montrul and Bowles, 2009; Montrul and Sánchez Walker, 2013).

^12^One reviewer queried whether or not subtle differences, as we have uncovered herein, in bilingual grammars would serve as a potential catalyst for changing the representational structure of monolingual grammars. It is outside of the scope of this paper to make such claims, not the least because it is difficult to find monolinguals of Catalan in particular to test what we would claim. That said, a general discussion on the matter is perhaps warranted. As monolinguals are in contact with bilinguals, especially in situations like Catalonia where bilingualism is the default state— rural enclaves of monolingualism would likely have significant contact with bilinguals, in person or via media. It would, thus, make sense that bilingual innovations could result in changes to monolinguals via various paths. We will highlight one herein. In light of L1 attrition research (see Schmid and Köpke, 2017, for review), we know that native grammars can change over time. We also know from Iverson (2012) and Iverson and Miller (2017) that all domains of grammar—even narrow syntax—can be affected by shifting input over thresholds for L1 change over time. And so, contact with bilinguals over the lifespan can induce innovations—if the threshold is tipped. Changes in production as a result in monolingual grammars will likely affect how the next generation sets the grammatical system, as argued for monolingual L1 acquisition (Lightfood, 1999) and heritage speakers (Pascual y Cabo and Rothman, 2012; Bayram et al., 2018), albeit via somewhat distinct provenance. Our results would be compatible with the argumentation in Perpiñán (2018) that specific context of Catalan-Spanish bilingualism is leading to language change and to the creation of a new variety of Catalan that allows for the optionality seen in our participants.
